# Brain ischemia downregulates the neuroprotective GDNF-Ret signaling by a calpain-dependent mechanism in cultured hippocampal neurons

**DOI:** 10.1038/cddis.2014.578

**Published:** 2015-02-12

**Authors:** M Curcio, I L Salazar, A R Inácio, E P Duarte, L M T Canzoniero, C B Duarte

**Affiliations:** 1 CNC-Center for Neuroscience and Cell Biology, University of Coimbra, Coimbra 3004-504, Portugal; 2 Department of Science and Technology, University of Sannio, Benevento 82100, Italy; 3 Doctoral Programme in Experimental Biology and Biomedicine, Center for Neuroscience and Cell Biology, University of Coimbra, Coimbra, Portugal; 4 Institute for Interdisciplinary Research, University of Coimbra (IIIUC), Coimbra, Portugal; 5 Wallenberg Neuroscience Center, Lund University, Lund 221 84, Sweden; 6 Department of Life Sciences, University of Coimbra, Coimbra 3004-517, Portugal

## Abstract

The glial cell line-derived neurotrophic factor (GDNF) has an important role in neuronal survival through binding to the GFR*α*1 (GDNF family receptor alpha-1) receptor and activation of the receptor tyrosine kinase Ret. Transient brain ischemia alters the expression of the GDNF signaling machinery but whether the GDNF receptor proteins are also affected, and the functional consequences, have not been investigated. We found that excitotoxic stimulation of cultured hippocampal neurons leads to a calpain-dependent downregulation of the long isoform of Ret (Ret51), but no changes were observed for Ret9 or GFR*α*1 under the same conditions. Cleavage of Ret51 by calpains was selectively mediated by activation of the extrasynaptic pool of N-methyl-d-aspartate receptors and leads to the formation of a stable cleavage product. Calpain-mediated cleavage of Ret51 was also observed in hippocampal neurons subjected to transient oxygen and glucose deprivation (OGD), a model of global brain ischemia, as well as in the ischemic region in the cerebral cortex of mice exposed to transient middle cerebral artery occlusion. Although the reduction of Ret51 protein levels decreased the total GDNF-induced receptor activity (as determined by assessing total phospho-Ret51 protein levels) and their downstream signaling activity, the remaining receptors still showed an increase in phosphorylation after incubation of hippocampal neurons with GDNF. Furthermore, GDNF protected hippocampal neurons when present before, during or after OGD, and the effects under the latter conditions were more significant in neurons transfected with human Ret51. These results indicate that the loss of Ret51 in brain ischemia partially impairs the neuroprotective effects of GDNF.

Neuronal injury induced by brain ischemia is partly owing to an excessive release of excitatory amino acids followed by toxic overactivation of glutamate receptors (excitotoxicity).^1, 2, 3^ The subsequent increase in [Ca^2+^]_i_ activates Ca^2+^-dependent proteolytic enzymes (e.g., calpains), which contribute to ischemic neurodegeneration.^4, 5, 6^ The extrasynaptic N-methyl-d-aspartate receptors (NMDAR) for glutamate are preferentially coupled to the activation of excitotoxic signaling mechanisms, including the activation of calpains, and were therefore suggested to have a key role in neuronal death.^7, 8, 9^

The glial cell line-derived neurotrophic factor (GDNF) protects neurons from excitotoxicity-induced cell death^10, 11, 12, 13^ and from ischemic damage.^14, 15, 16^ GDNF dimers bind to GFR*α*1 (GDNF family receptor alpha-1) with a high affinity, and this complex signals through the transmembrane Ret receptor tyrosine kinase.^17, 18^ The Ret receptor pre-mRNA is alternatively spliced in three isoforms that differ in the composition and length of the C-terminus tail. Once activated by the complex GDNF–GFR*α*1, Ret isoforms undergo transphosphorylation on several tyrosine residues, activating the Ras/mitogen-activated protein kinase (MAPK) and the phosphatidylinositol-3-kinase/AKT, responsible for cell survival or differentiation, and the phospholipase C-*γ* (PLC*γ*) pathway.^19, 20, 21, 22, 23^

Cerebral ischemia is known to alter the expression of the GDNF signaling machinery.^16^ Thus, the GDNF mRNA and protein were both found to be upregulated in experimental models of transient focal and global ischemia.^16, 24, 25, 26^ An increased expression of the mRNA for GFR*α*1^24, 27^ and Ret^27, 28^ was observed in damaged areas after transient middle cerebral artery occlusion (MCAO), a model of focal ischemia, and an upregulation of GFR*α*1 mRNA was also observed in the hippocampus following transient global ischemia in rats.^26^ However, whether GFR*α*1 and Ret protein levels are altered in transient brain ischemia, the impact on their signaling activity and the functional consequences, have not been investigated. In this work, we show that the Ret51 protein is selectively downregulated following excitotoxic injury and in brain ischemia, by a calpain-dependent mechanism, with a consequent decrease in GDNF signaling activity. This loss of Ret51 receptors impairs the neuroprotective effects of GDNF after transient *in vitro* ischemia.

## Results

### Ret51 is downregulated after excitotoxic stimulation

**Figure 1:**
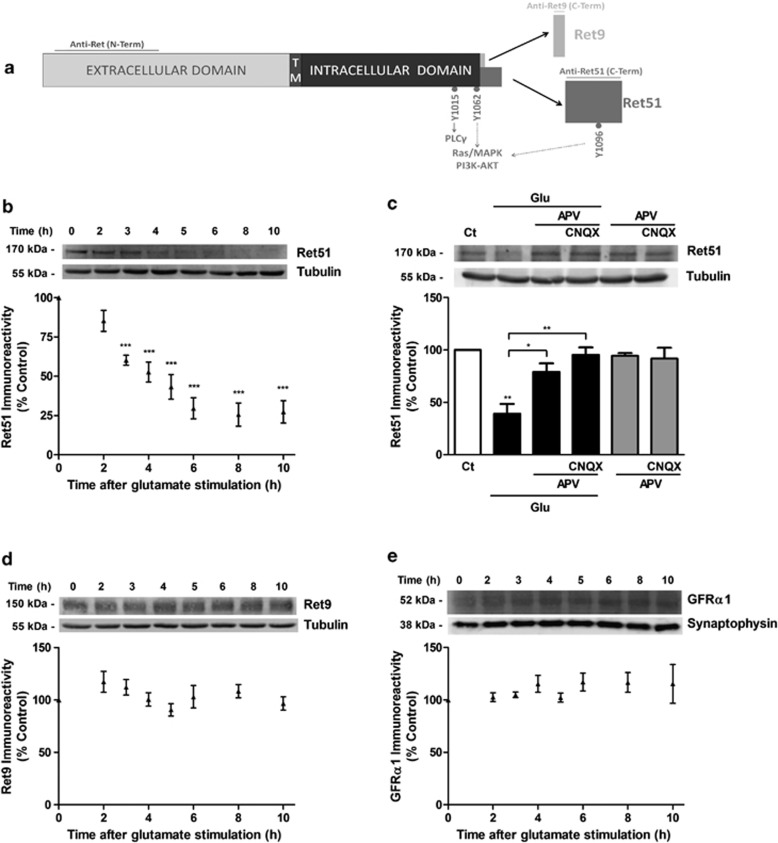
Ret51 isoform is selectively downregulated in hippocampal neurons subjected to excitotoxic stimulation by activation of NMDAR. (**a**) Alternative splicing of Ret transcripts gives rise to Ret51 and Ret9 isoforms, which differ in their C-terminus tail. The signaling pathways activated by the two isoforms are also indicated. Horizontal lines indicate the epitopes recognized by the anti-Ret antibodies used in this work: Ret (N-terminus) common to both isoforms, Ret51 (C-terminus) and Ret9 (C-terminus); TM: transmembrane domain. (**b**, **d**, **e**) Cultured hippocampal neurons (7 DIV) were subjected to excitotoxic stimulation with glutamate (125 *μ*M glutamate, 20 min) and further incubated in culture-conditioned medium for 2–10 h. Cell extracts were analyzed by western blotting using anti-Ret51 (**b**) or anti-Ret9 (**d**) antibodies, or with an anti-GFR*α*1 (**e**) antibody, at the indicated time points after excitotoxic stimulation. The ratio between Ret51 (**b**), Ret9 (**d**) and GFR*α*1 (**e**), and the loading control (tubulin (**b**–**d**) or synaptophysin (**e**)) was calculated, and the results are expressed as percentage of the control Ret51, Ret9 and GFR*α*1 protein levels. (**c**) Hippocampal neurons were pre-incubated (30min) with the glutamate receptors antagonists (100 *μ*M APV and 20 *μ*M CNQX) before and during glutamate (Glu) stimulation (125 *μ*M, 20 min). The cells were further incubated in culture-conditioned medium for 8 h in presence of the antagonists and extracts were analyzed by western blotting with an anti-Ret51 antibody. The ratio between Ret51 and the loading control (tubulin) was calculated, and the results are expressed as percentage of the control Ret51 protein levels. The results are the average±S.E.M. of 3–6 different experiments performed in independent preparations. Statistical analysis was performed using one-way ANOVA followed by (**b**, **d**, **e**) Dunnett's comparison test performed for each condition as compared with the control, not exposed to excitotoxic conditions (***P<0.001; Ret9 and GFR*α*1 protein levels after excitotoxic stimulation were not significantly different from the control), or by (**c**) Bonferroni's multiple comparison test (**P<0.01; *P<0.05, compared with the control condition or for the indicated comparisons)

To investigate the effects of excitotoxic stimulation on the expression levels of the proteins that assemble to form functional GDNF receptors, rat hippocampal neurons (7 days *in vitro* (DIV)) were challenged with glutamate (125 *μ*M, 20 min) and further incubated in culture-conditioned medium for different time periods. Under these conditions, glutamate evokes 40–50% of apoptotic-like cell death.^6, 29^ The effect of excitotoxic stimulation on the protein levels of GFR*α*1 and Ret isoforms was determined by western blotting (intracellular epitope of Ret51 and Ret9; Figure 1a). Glutamate stimulation downregulated the expression of Ret51 in a time-dependent manner, with a *t*_1/2_ of 4.09 h (Figure 1b), and maximal effects were observed at 8–10 h after the insult (Ret51 decreased to ~25% of the control). No significant changes were observed in Ret9 and in GFR*α*1 protein levels under the same conditions (Figures 1d and e).

To understand the role of glutamate receptor subtypes in the downregulation of Ret51 under excitotoxic conditions, we tested the effect of APV (DL-2-amino-5-phosphonovaleric acid) and CNQX (6-cyano-7-nitroquinoxaline-2,3-dione) that block NMDA and AMPA/kainate receptors, respectively. Incubation with APV alone reduced the glutamate-evoked downregulation of Ret51 to ~80% of the control, and co-incubation with APV and CNQX fully blocked the effect of excitotoxic stimulation on total Ret51 protein levels (Figure 1c).

### Ret51 is cleaved after excitotoxic stimulation by a calpain-dependent mechanism

**Figure 2:**
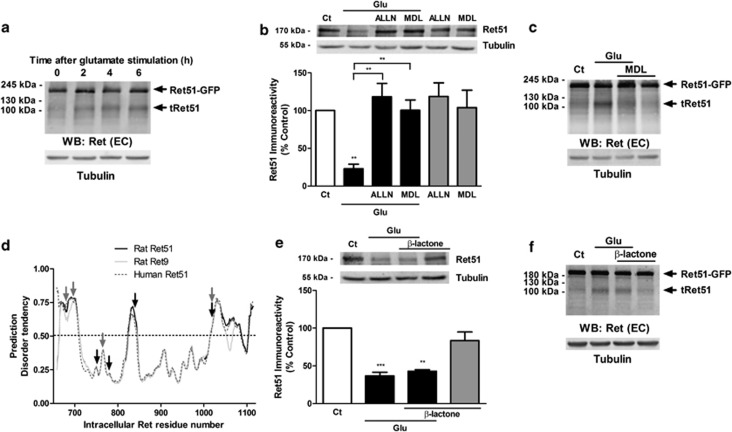
Ret51 is cleaved by calpain under excitotoxic conditions. (**a**) Cultured hippocampal neurons were transfected with hRet51-GFP and stimulated with glutamate (125 *μ*M, 20 min), followed by incubation in culture-conditioned medium for 2–6 h. Cell extracts were analyzed by western blot using an antibody against an EC epitope of Ret (Figure 1a). The results are representative of two independent experiments performed in different preparations. (**b**) Cultured hippocampal neurons (7 DIV) were pre-incubated (2 h) with the calpain inhibitors MDL28170 (MDL; 50 *μ*M) or ALLN (50 *μ*M), before and during glutamate stimulation (Glu; 125 *μ*M, 20 min). The cells were further incubated in culture-conditioned medium for 8 h with/without calpain inhibitors, and extracts were analyzed by western blotting with anti-Ret51 antibody, which interacts with an intracellular epitope. (**c**) The effect of the calpain inhibitor MDL28170 on glutamate-induced Ret51 cleavage was tested in cells transfected with hRet51-GFP. The cells were pre-incubated (2 h) with the calpain inhibitor MDL28170 (MDL; 50 *μ*M) before and during glutamate stimulation (Glu; 125 *μ*M, 20 min), and were further incubated in culture-conditioned medium for 6 h with/without calpain inhibitor. Cell extracts were analyzed by western blotting using an antibody against an EC epitope of Ret. The results are representative of two independent experiments performed in different preparations. (**d**) Prediction of the disorder tendency in the intracellular domain of rat Ret51 (black line, amino acids 659–1115), rat Ret9 (light gray line, amino acids 659–1173) and human Ret51 (dark gray dashed line, amino acids 658–1114) based on a bioinformatic analysis of the amino acid sequence of the proteins. Arrows point to the predicted calpain cleavage sites in human Ret51 (gray arrows: phenylalanine 676, serine 686, asparagine 763 and alanine 1019) or in rat Ret9/51 (black arrows: lysine 748, leucine 774, serine 837 and alanine 1020) identified with the GPS-CCD program. (**e**, **f**) The effect of the proteasome inhibitor *clasto*-lactacystin *β*-lactone (*β*-lactone; 1 *μ*M) was tested in hippocampal neurons, transfected (**f**) or not (**e**) with hRet51-GFP, by pre-incubating hippocampal neurons for 30 min with the drug before glutamate (Glu) stimulation. After excitotoxic stimulation, the cells were incubated in culture-conditioned medium with/without the inhibitor for 8 h. Extracts were analyzed by western blotting with an antibody against an intracellular epitope of Ret51 (**e**) or against the N-terminus of Ret (**f**). The results in **f** are representative of two independent experiments performed in different preparations. (**b**, **e**) The ratio between Ret51 and the loading control (tubulin) was calculated and the results were expressed as percentage of the control Ret51 protein levels. The results are the average±S.E.M. of 3–5 different experiments performed in independent preparations. Statistical analysis was performed using one-way ANOVA followed by Bonferroni's multiple comparison test (***P<0.001, **P<0.01 as compared with the control or for the indicated comparisons)

To determine the mechanisms (degradation/cleavage) inducing Ret51 downregulation, we performed complementary western blot experiments using an antibody against an extracellular (EC) epitope of Ret (Figure 1a) and cultured hippocampal neurons transfected with the human Ret51 fused intracellularly to green fluorescent protein (GFP; hRet51-GFP). Although the antibody did not detect the rat Ret9/51, a band corresponding to an apparent molecular weight of ~195 kDa was detected in hippocampal neurons transfected with hRet51-GFP (Figure 2a). Excitotoxic stimulation of hippocampal neurons transfected with hRet51-GFP induced a time-dependent accumulation of a cleavage product migrating at ~115 kDa (Figure 2a), containing the EC domain of the receptor, suggesting that the protein is cleaved in the intracellular region. Using an anti-GFP antibody, we did not detect the cleavage product containing GFP (not shown), suggesting that following excitotoxic stimulation with glutamate the intracellular fragment of Ret51 is degraded.

To determine the role of calpains in Ret51 downregulation in hippocampal neurons subjected to excitotoxic stimulation, the cells were incubated with glutamate in the presence or in the absence of the calpain inhibitors ALLN (N-acetyl-Leu-Leu-Norleu-al) or MDL28170 (carbobenzoxy-valinyl-phenylalaninal). Both inhibitors abrogated glutamate-evoked downregulation of Ret51 (Figure 2b). Furthermore, MDL28170 inhibited the cleavage of hRet51-GFP in transfected hippocampal neurons subjected to excitotoxic injury (Figure 2c). These results indicate that excitotoxic stimulation induces Ret51 cleavage through calpain activation.

Using the GPS-CCD (group-based prediction system-calpain cleavage detector) program^30^ for prediction of calpain cleavage sites, we found that the sites with the highest score in the C-terminus of human and rat Ret proteins are the following: Asn763>Ala1019>Ala1020>Phe676>Lys748>Leu774>Ser686>Ser837 (arrows in Figure 2d). As it was proposed that calpains cleave their substrates in rather disordered segments of the proteins,^31^ we used the metaPrDOS bioinformatic tool to predict the disorder tendency along the intracellular domain of Ret (rat Ret9, rat Ret51, human Ret51) sequences.^32^ The results showed that Ret proteins are more disordered in four segments of their C-terminus (amino acids in the rat Ret51 sequence: 664–711, 824–845, 1015–1087 and 1104–1115; Figure 2d). The first, second and third segments include the predicted calpain cleavage sites. The data shown in Figures 2a and c, together with the bioinformatic analysis, suggest that the human Ret51 may be cleaved on Phenylalanine676, giving rise to a fragment (containing the N-terminus of the receptor) with an apparent molecular weight of 115 kDa (Figures 2a and c).

As Ret can be degraded by the proteasome,^33, 34^ we tested whether this pathway could also account for the downregulation of Ret51 in hippocampal neurons subjected to excitotoxic stimulation. Inhibition of the proteasome with *clasto*-lactacystin *β*-lactone (*β*-lactone) was without effect on the glutamate-evoked decrease in endogenous Ret51 (Figure 2e). Similarly, no effect was observed on the downregulation of hRet51-GFP in transfected hippocampal neurons subjected to excitotoxic injury (Figure 2f), ruling out a contribution of the ubiquitin-proteasome system in Ret51 cleavage. Control experiments showed a dose-dependent downregulation of the chymotrypsin-like activity of the proteasome and an upregulation in polyubiquitin-conjugated proteins in hippocampal neurons incubated with *β*-lactone (Supplementary Figure 1).

Although caspase-3 can also cleave Ret receptors,^35^ this is unlikely to contribute to the downregulation of Ret51 under excitotoxic stimulation with glutamate given the low activity of the protease under these conditions^29^ (authors unpublished observations) and the effect of calpain inhibitors, which fully abrogated the glutamate-evoked downregulation of Ret51 (Figures 2b and c).

### Ret51 is targeted by calpain following activation of extrasynaptic NMDAR

**Figure 3:**
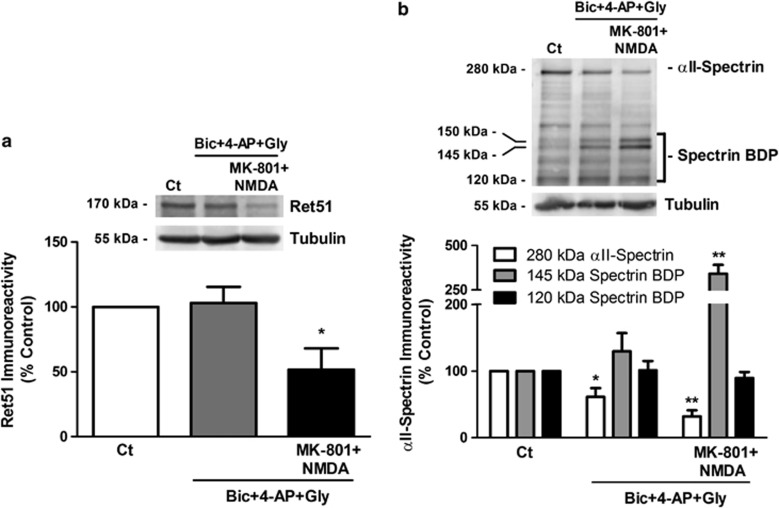
Ret51 is specifically cleaved by calpain following activation of extrasynaptic NMDAR. Cultured hippocampal neurons (15 DIV) were subjected to pharmacological protocols to activate synaptic or extrasynaptic NMDAR and were further incubated in culture-conditioned medium for 8 h. To induce synaptic activity the cells were treated with 50 *μ*M bicuculline (bic), 2.5 mM 4-AP and 10 *μ*M glycine (Gly) for 20 min. Selective activation of extrasynaptic NMDAR was performed after blocking the synaptic receptors with 50 *μ*M bicuculline, 2.5 mM 4-AP, 10 *μ*M glycine and 10 *μ*M MK-801 for 5 min. Extrasynaptic NMDAR were then stimulated for 20 min with 100 *μ*M NMDA in the absence of MK-801. Cell extracts were analyzed by western blotting with (**a**) anti-Ret51 (intracellular epitope) or (**b**) anti-*α*II-spectrin antibodies. In the latter condition, the immunoreactivity of the full-length protein (280 kDa *α*II-spectrin) and the cleavage products (SBDP) with 145 and 120 kDa was analyzed. The ratio between Ret51 protein levels (**a**) or intact/cleaved spectrin (**b**) and the loading control (tubulin) was calculated, and the results are expressed as percentage of the control. The results are the average±S.E.M. of 3–4 different experiments performed in independent preparations. Statistical analysis was performed using one-way ANOVA followed by Dunnett's test, comparing all the conditions with the control (**P<0.01; *P<0.05)

Activation of extrasynaptic NMDAR is selectively coupled to the induction of intracellular mechanisms distinct from those triggered by synaptic receptors.^8, 36^ As activation of NMDAR is specifically linked with Ret51 cleavage due to calpain activation (Figure 1c), we examined the differential effects of synaptic and extrasynaptic NMDAR activation on Ret51. In control experiments, we analyzed the activation of calpains under both conditions by measuring the cleavage of *α*II-spectrin, a well-known calpain substrate.^37^ Cleavage of *α*II-spectrin by calpains gives rise to a cleavage product with 145 kDa, whereas cleavage by caspase-3 accounts for the production of a 120 kDa fragment, and both proteases contribute to the production of a spectrin breakdown product (SBDP) with 150 kDa.^37^ Selective activation of extrasynaptic NMDAR significantly induced Ret51 and *α*II-spectrin cleavage, and accumulation of 145kDa SBDP. In contrast, activation of synaptic NMDAR showed a small effect on *α*II-spectrin cleavage, which was accompanied by a low accumulation of a 145 kDa SBDP and no significant Ret51 cleavage (Figure 3). As expected, there was no effect on the accumulation of the caspase-3-generated 120 kDa SBDP (Figure 3b).

### Ret51 is downregulated in an *in vitro* model of cerebral ischemia by a calpain-dependent mechanism following activation of glutamate receptors

**Figure 4:**
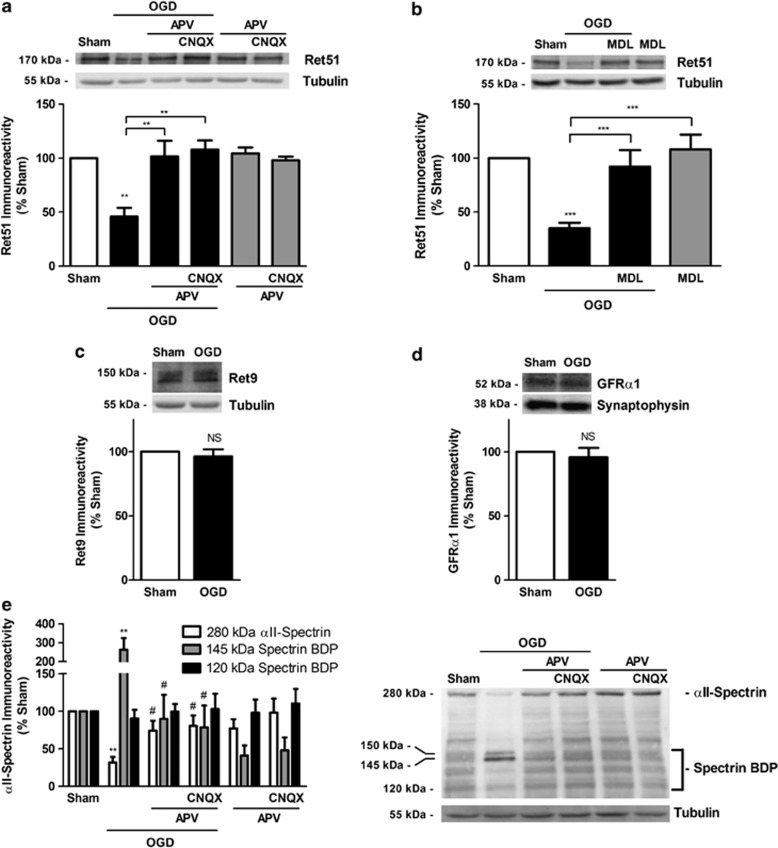
Ret51 is downregulated in *in vitro* ischemia by a calpain-dependent mechanism following activation of NMDAR. Cultured hippocampal neurons (15 DIV) were exposed to 90 min sham/OGD, followed by incubation in culture-conditioned medium for 8 h. (**a**, **b**, **e**) Where indicated, cells were pre-incubated for 30 min with the glutamate receptors antagonists (100 *μ*M APV and 20 *μ*M CNQX), or for 2 h with the calpain inhibitor MDL28170 (MDL; 50 *μ*M), and the drugs were also present throughout the experiment. Cell extracts were analyzed by western blotting with anti-Ret51 (intracellular epitope) (**a**, **b**), anti-Ret9 (**c**), anti-GFR*α*1 (**d**) or anti-*α*II-spectrin (**e**) antibodies. In the latter condition, the immunoreactivity of the full-length protein (280 kDa *α*II-spectrin) and the cleavage products (SBDP) with 145 or 120 kDa was analyzed. The ratio between Ret51 (**a**, **b**), Ret9 (**c**), GFR*α*1 (**d**), intact/cleaved *α*II-spectrin (**e**) and the loading control (tubulin (**a**, **b**, **c**, **e**) or synaptophysin (**d**]) was calculated, and the results are expressed as percentage of the control protein levels. The results are the average±S.E.M. of 3–5 different experiments performed in independent preparations. Statistical analysis was performed using one-way ANOVA followed by Bonferroni's multiple comparison test (**a**, **b**, **e**) or Student's *t*-test (**c**, **d**). (NS, not significant, ***P<0.001, **P<0.01, as compared with the sham condition or for the indicated comparisons; ^#^*P*<0.05 as compared with OGD condition)

We have previously shown a role for glutamate receptors in hippocampal neuronal death following oxygen and glucose deprivation (OGD), an *in vitro* model of transient global ischemia.^1^ Therefore, we compared the alterations in Ret51, Ret9 and GFR*α*1 protein levels in cultured hippocampal neurons subjected to OGD with those observed after excitotoxic stimulation. Transient incubation of rat hippocampal neurons (15 DIV) under OGD for 90 min induces ~35% cell death when determined 7–12 h after the insult.^1, 4^ The results showed a downregulation of Ret51 protein levels to ~35% of the sham, whereas the Ret9 and GFR*α*1 were not affected (Figures 4a–d). Incubation of hippocampal neurons with APV alone or in combination with CNQX completely blocked OGD-induced downregulation of Ret51 (Figure 4a).

To determine whether calpain activation also has a role in Ret51 downregulation in hippocampal neurons subjected to OGD, we tested the effect of MDL28170. The calpain inhibitor significantly reduced the OGD-induced loss of Ret51 protein levels to ~90% of the control, showing a key role for calpains in the downregulation of the receptor (Figure 4b). As expected, OGD significantly reduced the full-length spectrin protein levels, giving rise to a consistent accumulation of the 145 kDa SBDP formed upon cleavage by calpains (Figure 4e). Inhibition of the NMDAR with APV, in the absence or in the presence of the AMPA/kainate receptor inhibitor CNQX, completely prevented the cleavage of the full-length protein and the accumulation of the 145 kDa SBDP (Figure 4e). No significant changes were observed in the caspase-3-generated 120 kDa SBDP, indicating a minor contribution of caspase-3 to spectrin cleavage after OGD.

### Ret isoforms are downregulated in *in vivo* ischemia

**Figure 5:**
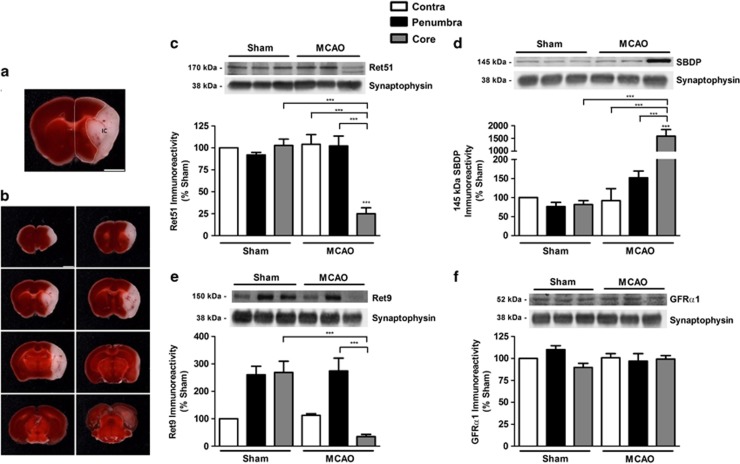
Ret isoforms are downregulated in *in vivo* ischemia. (**a**) Representative image of the regions dissected from the ipsilateral brain hemisphere of C57BL/6 mice subjected to 45 min sham surgery or MCAO, considered as infarct core (IC) and penumbra (delineated). Scale bars, 2 mm. (**b**) Representative image of the cerebral infarct core following a transient (45 min) occlusion of the MCA, in C57BL/6 mice, as given by lack of 2,3,5-triphenyltetrazolium chloride staining in contiguous 1-mm-thick coronal slices (white). (**c**–**f**) Adult C56BL/6 mice were subjected to transient 45 min MCAO/sham, and Ret51 (**c**), 145 kDa SBDP (**d**), Ret9 (**e**) and GFR*α*1 (**f**) protein levels were determined in the infarct core, penumbra and contralateral cortex 48 h after the lesion by western blotting. The ratio between Ret51 (**c**), 145 kDa SBDP (**d**), Ret9 (**e**) and GFR*α*1 (**f**) protein levels, and the loading control (synaptophysin) was calculated, and the results obtained in the contralateral hemisphere of sham-operated mice were set to 100%. The results are the average±S.E.M. of 3–4 independent experiments performed in different animals. Statistical analysis was performed using one-way ANOVA followed by Bonferroni's multiple comparison test (***P<0.001, as compared for the indicated comparisons). The results obtained for GFR*α*1 are not statistically different

In additional experiments, we determined the alterations in the levels of GDNF receptors in the brain after transient MCAO, a model of focal brain ischemia.^38, 39^ Adult mice were subjected to 45min occlusion of the right middle cerebral artery (MCA), and extracts were prepared from the ischemic core and the penumbra region of the ipsilateral brain hemisphere, as well as from the contralateral (contra) brain hemisphere (Figures 5a and b), 48 h after the lesion. At this time point, the brain infarct is fully developed and is not expected to further increase.^40^ In sham-operated mice, protein extracts were prepared from equivalent brain regions. A marked decline of protein levels of both Ret isoforms (to ~25% and ~35% for Ret51 and Ret9, respectively, as compared with the contralateral region of sham-operated mice) was observed in the ischemic core, whereas no significant changes were observed in the penumbra region and in the contralateral side in MCAO-operated mice (Figures 5c and e). Interestingly, in the core region, the downregulation of the mature form of Ret51, with 170 kDa, was accompanied by an increase in the abundance of a 150 kDa protein that is likely to correspond to an immature form of the protein present in the endoplasmic reticulum.^41, 42^ These results show a comparable pattern of change in Ret51 protein levels following excitotoxic stimulation (Figures 1b and c) and in both ischemia models used in this work (Figures 4a and b, and Figure 5c). GFR*α*1 protein levels did not change significantly in the brain of mice subjected to MCAO (Figure 5f).

Given the role of calpains in the downregulation of Ret51 in hippocampal neurons subjected to OGD and to excitotoxic stimulation, we checked for the accumulation of the 145 kDa SBDP in the core and penumbra regions of mice subjected to 45 min of MCAO. As expected, there was a strong increase in the immunoreactivity in the ischemic core, showing an upregulation of calpain activity, whereas the accumulation of the 145 kDa SBDP in the penumbra region was not statistically significant (Figure 5d). Taken together, these results suggest that calpain activation may also contribute to the downregulation of Ret51 in the core region in the brain cortex of mice subjected to MCAO.

The downregulation of Ret9 in the core region after transient MCAO (Figure 5e) contrasts with the results obtained under excitotoxic conditions (Figure 1d) or in *in vitro* ischemia (Figure 4c). This may be due to (i) the stronger injury induced by *in vivo* ischemia when compared with the *in vitro* models, which may further upregulate calpain activity, and/or (ii) downregulation of Ret9 in astrocytes (present in low amounts in hippocampal cultures), as these receptors are also expressed in this cell type.^11^

### GDNF-dependent signaling is downregulated under excitotoxic conditions and brain ischemia

Ret51 downregulation observed under excitotoxic conditions and in two different models of brain ischemia is likely to have a significant impact in the signaling activity of the intact full-length receptor. To address this question, we analyzed (i) Ret51 phoshorylation on Tyrosine-1062 (present in Ret51 and Ret9), (ii) PLC*γ*1 phosphorylation, as it follows the interaction of the enzyme with pTyr1015 in the active receptor^19^ and (iii) ERK phosphorylation, as it is a downstream mediator of the signaling cascade activated by pTyr1062 (present in Ret51 and Ret9) and pTyr1096 (present only in Ret51; Figure 1a).^20, 21, 33^

In preliminary experiments, we tested the effect of excitotoxic injury on GDNF (10 ng/ml)-induced phosphorylation of Ret on Tyr1062, which is coupled to the activation of neuronal survival pathways (Figure 1a).^16, 20, 21, 23^ These studies were performed in hippocampal neurons cultured for 15 DIV that show a higher phosphorylation of Ret under resting conditions and a more robust response to GDNF than 7 DIV neurons (Supplementary Figure 2). Incubation with GDNF was performed during 10 min, which induces maximal phosphorylation of the receptors (not shown). As hippocampal neurons cultured for 15 DIV are more sensitive to excitotoxic injury than younger cultures, the concentration of glutamate in these experiments was reduced to 50 *μ*M. Under these conditions, Ret51 protein levels were downregulated following excitotoxic stimulation, in a time- and calpain-dependent manner, with a t_1/2_ of 6.80 h, whereas Ret9 expression was not affected (Supplementary Figure 2), similarly to the results obtained in 7 DIV neurons (Figures 1 and 2).

**Figure 6:**
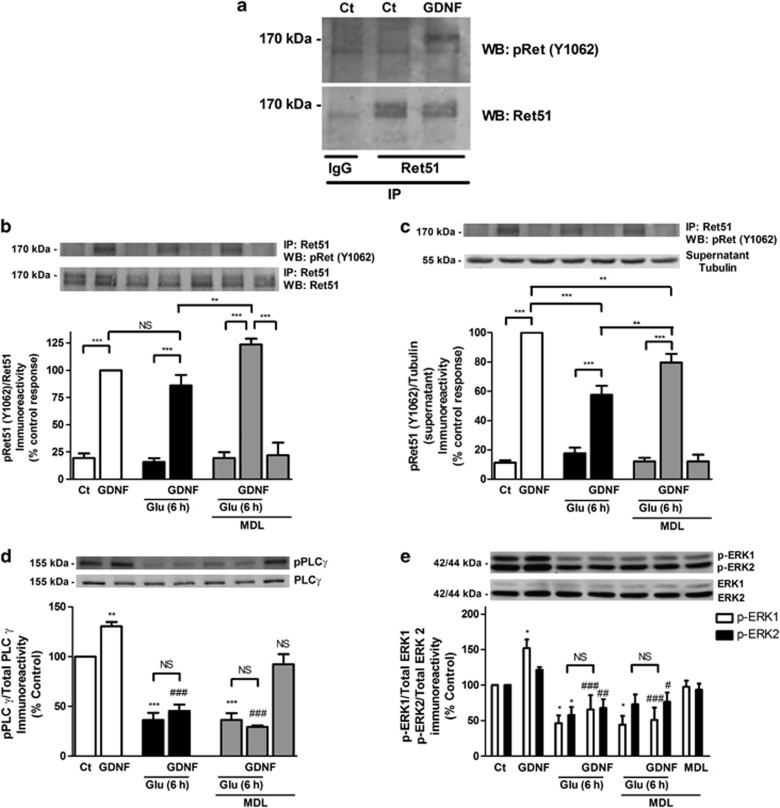
Ret51-dependent signaling is downregulated under excitotoxic conditions. (**a**) Cultured hippocampal neurons (15 DIV) were stimulated with GDNF (GDNF, 10 ng/ml) or maintained under control conditions for 10 min and lysed, and Ret51 proteins were immunoprecipitated. As control condition, normal goat IgG was used. The immunoprecipitates were analyzed by western blotting using anti-phospho-specific Ret (pRet, Y1062) and anti-Ret51 antibodies. The supernatants from the immunoprecipitation were subjected to western blot analysis with an anti-tubulin antibody to confirm that equal amounts of protein were analyzed. (**b**–**e**) Hippocampal neurons (15 DIV) were stimulated with 10 ng/ml GDNF (GDNF) under control conditions or after excitotoxic stimulation with glutamate (Glu; 50 *μ*M, 20 min). Under the latter experimental conditions, the cells were incubated in culture-conditioned medium for 6 h after the toxic insult before stimulation with GDNF. When the effect of the calpain inhibitor MDL28170 (MDL; 50 *μ*M) was tested, the cells were pre-incubated with the inhibitor for 2 h before glutamate stimulation, and the inhibitor was also present during all additional experimental manipulation. (**b**, **c**) Cell extracts were subjected to immunoprecipitation and western blot analysis as in **a**. The ratio between (**b**) phospho-Ret (pRet, Y1062) protein levels and total Ret51 immunoreactivity or (**c**) phospho-Ret (pRet, Y1062) and tubulin (in the supernatant) was calculated, and the results obtained in control cells stimulated with GDNF were set to 100% (**b**, **c**). (**d**, **e**) GDNF-induced downstream signaling under excitotoxic conditions was analyzed by western blot with anti-pPLC*γ*1 (**d**) or anti-pERK1/2 (**e**) antibodies, using total PLC*γ*1 and ERK1/2 as loading control, respectively. The ratio between the phospho- and the total protein levels was calculated, and the results obtained under resting conditions were set to 100%. (**b**–**e**) The results are the average±S.E.M. of 4–6 different experiments performed in independent preparations. Statistical analysis was performed using one-way ANOVA followed by Bonferroni's multiple comparison test. ((**b**,**c**): ***P<0.001, **P<0.01, as compared with the GDNF stimulated cells or for the indicated comparisons; (**d**,**e**): NS, not significant; ***P<0.001, **P<0.01, *P<0.05 as compared with the control or for the indicated comparisons; ^###^*P*<0.001, ^##^*P*<0.01 as compared with GDNF stimulated cells under control conditions)

The effect of excitotoxic injury on Ret51 phosphorylation (activation) was evaluated after immunoprecipitation of the receptor with an antibody specific for this Ret isoform. Figure 6 (panels a and b) shows that GDNF significantly increases Ret51 phosphorylation under control conditions, without affecting total Ret51 protein levels (Supplementary Figure 2). Excitotoxic stimulation with glutamate significantly inhibited the GDNF-induced total increase in pRet51 (Tyr1062) to ~58% of the control (Figure 6c), as determined 6h after excitotoxic stimulation, when Ret51 protein levels are reduced to ~50% of the control (Supplementary Figure 2). The effect of glutamate stimulation on total Ret51 phosphorylation was only partially reduced by calpain inhibition with MDL28170 (Figure 6c), which fully prevents the downregulation of the receptor (Supplementary Figure 2). These results provide an estimation of the effects of excitotoxic injury on the total number of active (phosphorylated) Ret51 receptors, which is related with the decrease in the total number of receptors. To determine whether the Ret51 receptors that are not affected by the toxic insult with glutamate are still likely to be activated (phosphorylated) following stimulation with GDNF, the alterations in pRet51 were expressed as a function of the total receptor levels in hippocampal neurons before and after injury, in the absence and in the presence of the calpain inhibitor (Figure 6b). The results show no decrease of the GDNF-induced pRet51/Ret51 ratio following excitotoxic injury, in the absence or in the presence of the calpain inhibitor, indicating that the receptor population remaining after injury can still be phosphorylated.

In addition to the effects of excitotoxic injury on Ret51 phosphorylation, there was also an impairment of GDNF-induced PLC*γ*1 phosphorylation (Figure 6d) and ERK activation (Figure 6e). Moreover, the activity of both signaling pathways was still downregulated when the experiments were conducted in the presence of the calpain inhibitor MDL28170, indicating that activation of GDNF receptors is no longer coupled to the stimulation of survival pathways.

**Figure 7:**
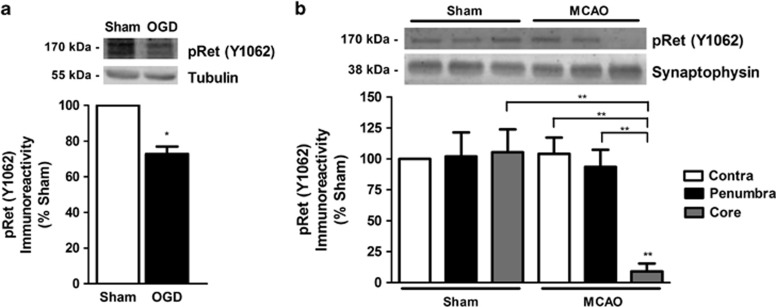
Phospho-Ret is downregulated in brain ischemia. (**a**) Cultured hippocampal neurons (15 DIV) were exposed to 90 min sham/OGD, followed by incubation in culture-conditioned medium for 8 h. Cell extracts were analyzed by western blotting with anti-phospho-specific anti-Ret (pRet, Y1062) antibody. The results presented are the ratio between phospho-Ret (pRet, Y1062) protein levels and the loading control (tubulin). Sham protein level was set to 100%. (**b**) Adult C56BL/6 mice were subjected to transient 45 min MCAO/sham. pRet (Y1062) protein levels were determined in the infarct core, penumbra and contralateral cortex 48 h after the lesion by western blotting. The ratio between pRet (Y1062) protein levels and the loading control (synaptophysin) was calculated, and the results obtained in the contralateral hemisphere of sham-operated mice were set to 100%. The results are the average±S.E.M. of four independent experiments performed in different preparations/animals. Statistical analysis was performed using Student's *t*-test (**a**) or one-way ANOVA followed by Bonferroni's multiple comparison test (**b**) (**P<0.01, *P<0.05 as compared for the indicated comparisons)

Given the calpain-mediated downregulation of Ret51 in excitotoxic and ischemic conditions, we investigated whether pRet downregulation was also observed in the *in vitro* and *in vivo* models of brain ischemia. Transient exposure of hippocampal neurons to OGD decreased the expression of pRet to ~70% of the sham (Figure 7a). A decrease in pRet was also observed in the infarct core after transient MCAO, but not in the penumbra region (Figure 7b). The reduction in pRet in the ischemic core after *in vivo* ischemia may be related, at least in part, with the decrease in total Ret51 and Ret9 protein levels, which contrasts with the stability in the receptor levels in the penumbra region (Figures 5a and c).

### GDNF protects hippocampal neurons from excitotoxic stimulation and *in vitro* brain ischemia

**Figure 8:**
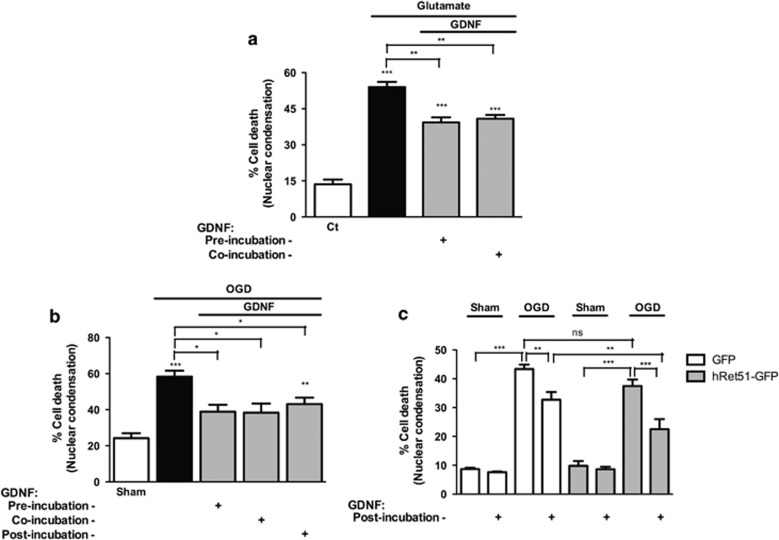
Neuroprotection by GNDF under excitotoxic conditions and *in vitro* ischemia. (**a**) Cultured hippocampal neurons (15 DIV) were challenged with 50 *μ*M glutamate for 20 min and where indicated they were incubated with 10 ng/ml GDNF (GDNF) during excitotoxic stimulation (co-incubation), or pre-incubated with the neurotrophic factor for 30 min (pre-incubation). (**b**) Cultured hippocampal neurons (15 DIV) were exposed to sham/OGD for 90 min and where indicated they were incubated with 10 ng/ml GDNF (GDNF) during the insult (co-incubation), immediately after (post-incubation) or pre-incubated with the neurotrophic factor for 30 min (pre-incubation). When tested, the neurotrophic factor was also present during the incubation period in culture-conditioned medium that followed glutamate stimulation (**a**) or sham/OGD (**b**). Cell death was assessed 8 h after excitotoxic stimulation (**a**) or 12 h after sham/OGD (**b**) by fluorescence microscopy, using the fluorescent dye Hoechst 33342. (**c**) Hippocampal neurons were transfected with GFP or hRet51-GFP and exposed to sham/OGD for 90 min. After the insult, the cells were further incubated in culture-conditioned medium for 12 h. When tested, 10 ng/ml GDNF (GDNF) was added to the cells immediately after the insult. The transfected cells were identified by immunocytochemistry with an anti-GFP antibody, and the viability of GFP or hRet51-GFP-transfected cells was evaluated with Hoechst 33342. The results are the average±S.E.M. of 3–7 different experiments performed in independent preparations. Statistical analysis was performed using one-way ANOVA followed by Bonferroni's multiple comparison test (***P<0.001, **P<0.01, *P<0.05 as compared with the control/sham cells or for the indicated comparisons)

The downregulation of Ret51 after excitotoxic injury in cultured hippocampal neurons and in brain ischemia may affect the neuroprotective signaling induced by GDNF. To investigate the neuroprotective effects of GDNF in cultured hippocampal neurons, we first compared the effect of GDNF added to hippocampal neurons before (30 min) or during the excitotoxic insult. Hippocampal neurons (15 DIV) were incubated with 50 *μ*M glutamate for 20 min, in presence or not of GDNF, and were further incubated in culture-conditioned medium for 8 h (Figure 8a). Under these conditions, excitotoxic stimulation induced ~50% of cell death, and incubation with the neurotrophic factor for 30min before or during the insult reduced neuronal death by ~15%.

We also investigated the putative neuroprotective effects of GDNF in OGD. Incubation with GDNF for 30 min before or during the ischemic insult significantly reduced cell death by ~20%. Importantly, when GDNF was added immediately after OGD, there was also a protective effect of ~15% (Figure 8b). The protective effect of GDNF under the latter conditions may be, at least in part, mediated by Ret51, but the downregulation of the receptor after OGD may compromise the neuroprotective activity of GDNF. To address this question, we compared the effects of GDNF in hippocampal neurons transfected with GFP or with hRet51-GFP. In these experiments, the cells were incubated with GDNF during the period after the insult. OGD increased the rate of cell death to 43% in hippocampal neurons transfected with GFP, and under these conditions GDNF significantly decreased cell death to 32% when added after the insult. In hippocampal neurons transfected with hRet51-GFP, the presence of GDNF during the incubation period after OGD further decreased cell death to 22%, suggesting that the downregulation of Ret51 receptors indeed decreases the neuroprotective effects of GDNF in hippocampal neurons subjected to *in vitro* ischemia (Figure 8c). As expected, cells expressing GFP exhibited a pattern of neuroprotection by GDNF similar to the one observed in non-transfected cells (Figure 8b).

## Discussion

In this study, we found that the GDNF co-receptor Ret51 is selectively downregulated in cultured hippocampal neurons subjected to excitotoxic conditions, and in *in vitro* brain ischemia, by a mechanism dependent on the activation of extrasynaptic NMDAR coupled to stimulation of calpains. The cleavage of Ret51 was also observed in the ischemic core after transient MCAO, and decreased the GDNF-induced signaling mechanisms and neuroprotection. Therefore, although the ischemic injury upregulates GDNF gene expression and protein levels in the injured brain region,^16, 24, 25, 26, 43^ this is less likely to provide an increased neuroprotection. In addition to Ret51, the TrkB receptors for BDNF are also downregulated under excitotoxic conditions and after transient MCAO,^6, 44^ suggesting that the impairment of the endogenous neuroprotective signaling mechanisms mediated by receptor tyrosine kinases may be a key event in neuronal death in brain ischemia.

Calpains are important mediators of ischemic neurodegeneration and of excitotoxic neuronal damage.^3, 5^ Combining *in vitro* pharmacological approaches and biochemical analysis, we have found that calpains have a key role in the downregulation of Ret51 under excitotoxic conditions and in hippocampal neurons subjected to OGD. The role of calpains in Ret51 cleavage is supported by the following pieces of evidence: (i) calpain inhibitors prevented the excitotoxicity- and OGD-induced downregulation of total Ret51; (ii) Ret51 cleavage under excitotoxic conditions and in the *in vitro* and *in vivo* models of brain ischemia was correlated with an increase in calpain activity; (iii) inhibition of calpains prevented the glutamate-evoked formation of a Ret51 cleavage product; and (iv) several putative calpain cleavage sites, located in intracellular sequences of Ret51, were identified by the GPS-CCD program. Interestingly, the glutamate-induced cleavage of Ret51 was specifically mediated by activation of extrasynaptic NMDAR, and these results were correlated with the preferential coupling of this population of glutamate receptors to the upregulation of calpain activity. Previous studies have shown that extrasynaptic NMDAR-coupled m-calpain activation is linked to neurodegeneration while the synaptic pool of NMDAR is coupled to neuroprotective activation of *μ*-calpain.^7, 9^

In contrast with the effects on Ret51, excitotoxic stimulation and OGD did not affect total Ret9 or GFR*α*1 protein levels in cultured hippocampal neurons, showing the specificity of the effects on Ret51. The differences in the C-terminus tail of Ret9 and Ret51 (Figures 1a and 2d), including in the disorder tendency, may account for the differential sensitivity to calpains. Alternatively, the selective activity of calpain on Ret51 may be owing to differences in the subcellular localization of the two Ret isoforms.^41^

Given the key role of excitotoxicity in OGD-induced downregulation of Ret51 and cell death, we investigated the effect of toxic stimulation with glutamate on GDNF-induced Ret51 activation by transphosphorylation. As expected, the downregulation of Ret51 was associated with a decrease in total receptor phosphorylation detected in the cells. However, calpain inhibition, which fully abrogates the downregulation of Ret51 under excitotoxic conditions, did not completely prevent the decrease in receptor phosphorylation, suggesting that additional mechanisms may contribute to the impairment of the GDNF-induced receptor activation. A decrease in the ATP levels in neurons following a toxic stimulation with glutamate^1^ may contribute to the impairment of the mechanisms of receptor activation by transphosphorylation. Although at least some of the cleaved Ret51 receptors remain in the cell after excitotoxic stimulation (Figure 2a), they are unlikely to have an important role as dominant negatives, to prevent the activation of the full-length Ret51 receptors, as the pRet51/total Ret51 in hippocampal neurons subjected to excitotoxic injury was not significantly different from the control. As Ret9 and Ret51 appear to form independent complexes following stimulation with GDNF,^33, 45^ the cleaved Ret51 receptors are also unlikely to modulate the signaling activity of Ret9 receptors by a dominant-negative type of effect.

The partial reduction of GDNF-induced Ret51 signaling in hippocampal neurons subjected to excitotoxic stimulation contrasts with a full inhibition of the two downstream pathways, PLC*γ* and ERK1/2. PLC*γ* is activated directly upon binding to the phosphorylated Ret receptors, while ERK1/2 activation requires the participation of several signaling intermediates.^20, 23, 46^ Furthermore, no protective effect was observed in the presence of the calpain inhibitor MDL28170, suggesting an overall impairment of the GDNF signaling mechanisms, which should prevent the neuroprotective signaling activity, even when the truncation of the receptors is blocked. In particular, the inhibition of the ERK pathway is likely to have a great impact in neuroprotection under excitotoxic conditions.^10, 11^

The neuroprotective effects of GDNF were tested in hippocampal neurons by incubation with the neurotrophic factor before, during and after OGD. In all experimental conditions, GDNF reduced cell death. However, when the experiments were performed in hippocampal neurons transfected with hRet51, the OGD-induced cell death was significantly reduced. These results show that indeed the time-dependent downregulation of Ret51 after OGD significantly decreases the neuroprotective effects of GDNF and may therefore contribute to the demise process in brain ischemia *in vivo*. The fact that not all Ret51 protein is cleaved/degraded after the ischemic insult may explain why GDNF treatment immediately after 90 min MCAO in rats still reduced infarct volume, apoptotic cell death and autophagic effects when determined at 24 h after the surgery.^15^ Moreover, 10 min intravenous application of TAT-GDNF immediately after 30 min MCAO reduced the number of caspase-3-immunoreactive and DNA-fragmented cells, and increased the number of viable neurons in the striatum, as found 3 days after the insult.^47^ The observed neuroprotective effects of GDNF might be mediated by at least four different mechanisms: (i) reduction in NMDA-mediated calcium currents,^11^ (ii) upregulation of antiapoptotic gene expression,^12^ (iii) suppression of free radical production^48^ and/or (iv) increase in neuronal metabolic activity.^49^

The observed downregulation of Ret51 after transient MCAO in mice (at *t*=48 h) contrasts with the effect of permanent MCAO in rats, which increases Ret51 protein levels in the ipsilateral region at 3–24 h.^50^ These contrasting results suggest that the effects of the ischemic injury on Ret51 protein levels depend on the type of insult or, alternatively, the differences may be owing to the distinct time points after the injury onset used in the two studies. The differential effects observed in the experiments using the permanent brain ischemia model and in cultured hippocampal neurons subjected to OGD may be owing to the contribution of glial cells, which also express Ret receptors^11^ and are present in low amounts in the hippocampal cultures.^4^

Together, the results show a role for calpains in the cleavage of Ret51 under excitotoxic conditions and in brain ischemia. Given the neuroprotective role of GDNF, even when tested after the ischemic injury, and the upregulation of GDNF in the ischemic brain, the results suggest that preserving Ret receptors may constitute a good strategy to increase the endogenous neuroprotective mechanisms. Such strategy may also be relevant under other conditions characterized by excitotoxic cell death, such as in cerebral trauma, epileptic seizures and chronic neurodegenerative disorders.

## Materials and Methods

### Chemicals

Neurobasal medium, gentamycin and Hoechst 33342 were purchased from Invitrogen (Paisley, UK). NeuroCult SM1 neuronal supplement was obtained from STEMCELL Technologies (Grenoble, France) and MDL28170, ALLN and *β*-lactone were obtained from Merck Millipore (Nottingham, UK). APV, CNQX, bicuculline, 4-aminopyridine (4-AP), (5*R*,10*S*)-(+)-5-methyl-10,11-dihydro-5*H*-dibenzo[*a*,*d*]cylcohepten-5,10-imine maleate (MK-801) and NMDA were obtained from Tocris (Bristol, UK). Protein G sepharose and the fluorescent substrate for alkaline phosphatase-based detection of protein blot (ECF) were purchased from GE Healthcare (Munich, Germany). Glutamine, kynurenic acid, phenylmethylsulfonyl fluoride (PMSF), chymostatin, leupeptin, antipain and pepstatin were obtained from Sigma-Aldrich (Sintra, Portugal) and the recombinant human GDNF was obtained from Peprotech (London, UK).

### Antibodies

The following primary antibodies were used: anti-Ret51 (sc-1290; 1:500), anti-Ret9 (sc-167; 1 : 200), anti-pRet (Tyr1062, sc-20252-R; 1 : 500), anti-Ret (N-term, sc-13104; 1 : 200) and anti-PLC*γ*1 (sc-81; 1 : 1 000) were purchased from Santa Cruz Biotechnology (Santa Cruz, CA, USA); anti-GFR*α*1 (AF560; 1 : 500) from R&D Systems (Abingdon, UK); anti-pPLC*γ*1 (SAB4300271; 1 : 500) from Sigma-Aldrich; anti-GFP (no. 598; 1 : 1000) from MBL International (Woburn, MA, USA); anti-ERK1/2 (no. 610030; 1 : 5000) from BD Biosciences (San Jose, CA, USA); anti-pERK1/2 (V803A; 1 : 500) from Promega (Madison, WI, USA); and anti-*α*II-spectrin from Merck Millipore (MAB1622; 1 : 1000). The anti-synaptophysin antibody (ab52636; 1 : 20,000) was purchased from Abcam (Cambridge, UK) and anti-*β*-tubulin from Sigma-Aldrich (T7816; 1 : 400 000). The antibody against calpain-mediated SBDP with 145 kDa (145-kDa SBDP; 1 : 500) was kindly provided by Dr. Ben Bahr (Biotechnology Research and Training Center, University of North Carolina Pembroke, Pembroke, North Carolina).^51^ The anti-rabbit and anti-mouse secondary antibodies conjugated with alkaline phosphatase were obtained from Jackson ImmunoResearch (Newmarket, UK), and the anti-goat antibody was from Santa Cruz Biotechnology or Jackson ImmunoResearch. The Alexa Fluor 488-conjugated goat anti-rabbit antibody used in immunocytochemistry experiments was obtained from Invitrogen.

### Hippocampal cultures

Primary cultures of rat hippocampal neurons were prepared from the hippocampi of E18–E19 Wistar rat embryos as previously described.^1^ Hippocampal neurons were plated on poly-d-lysine-coated surfaces and cultured in neurobasal medium supplemented with NeuroCult SM1 neuronal supplement (1 : 50 dilution), 25 *μ*M glutamate, 0.5 mM glutamine and 0.12 mg/ml gentamycin. For biochemical analysis, neurons were plated at a density of 90.0 × 10^3^ cells/cm^2^, whereas for morphological studies neurons were plated on coverslips at a density of 80.0 × 10^3^ cells/cm^2^. The cultures were kept at 37 °C in a humidified incubator with 5% CO_2_/95% air for 7 or 15 days (7/15 DIV).

### Glutamate treatment

Excitotoxic stimulation with glutamate was carried out by challenging 7 and 15 DIV hippocampal neurons with 125 or 50 *μ*M glutamate, respectively, for 20 min, and the cells were further incubated in culture-conditioned medium for the indicated periods of time. When appropriate, the cells were pre-incubated with the calpain inhibitor MDL28170 (50 *μ*M) or ALLN (50 *μ*M), with the glutamate receptors antagonists APV (100 *μ*M) or CNQX (20 *μ*M), with the proteasome inhibitor *β*-lactone (1 *μ*M) or with 10 ng/ml GDNF, as indicated in the figure captions. All compounds were also present during the stimulation and recovery periods. To evaluate Ret51 signaling activity, hippocampal neurons were incubated with 10 ng/ml GDNF for 10 min, under control conditions or 6 h after excitotoxic stimulation.

### Oxygen-glucose deprivation

OGD was performed by incubating hippocampal neurons (15 DIV) in a glucose-free saline buffer (116 mM NaCl, 25 mM sucrose, 10 mM HEPES, 5.4 mM KCl, 0.8 mM MgSO_4_, 1 mM NaH_2_PO_4_, 1.8 mM CaCl_2_ and 25 mM NaHCO_3_) in an anaerobic chamber with 10% H_2_, 85% N_2_ and 5% CO_2_ (Forma Anaerobic System, Thermo Fisher Scientific, Porto Salvo, Portugal) at 37 °C for 1.5 h. The OGD buffer was then replaced by a conditioned medium and the cultures were returned to the humidified 95% air/5% CO_2_ incubator for the indicated post-incubation time period. Under control conditions (Sham), the cells were incubated in the saline buffer described above, supplemented with 25 mM glucose instead of sucrose and kept in the humidified 95% air/5% CO_2_ incubator at 37 °C. When appropriate the cells were pre-incubated with the calpain inhibitor MDL28170 (50 *μ*M), with the glutamate receptors antagonists APV (100 *μ*M) or CNQX (20 *μ*M), or with 10 ng/ml GDNF, as indicated in the figure captions. In this case, all compounds were also present during the stimulation and recovery periods.

### Stimulation of synaptic *versus* extrasynaptic NMDAR

Activation of synaptic *versus* extrasynaptic NMDAR was performed as previously described,^1, 52^ in a basal saline buffer containing 132 mM NaCl, 4 mM KCl, 1.4 mM MgCl_2_, 2.5 mM CaCl_2_, 6 mM glucose and 10 mM HEPES. Synaptic NMDAR were stimulated by incubating hippocampal neurons (15 DIV) with 50 *μ*M bicuculline, 2.5 mM 4-AP and 10 *μ*M glycine for 20 min. To stimulate the extrasynaptic pool of NMDAR, hippocampal neurons (15 DIV) were incubated with 50 *μ*M bicuculline, 2.5 mM 4-AP, 10 *μ*M glycine and 10 *μ*M MK-801 for 5 min, and washed once with a similar solution but in the absence of MK-801 before incubation with 100 *μ*M NMDA for 20 min. Neurons were then allowed to recover in culture-conditioned medium for the indicated periods of time.

### Neuron transfection with calcium phosphate

The plasmid encoding the full-length human Ret51 fused C-terminally to enhanced GFP was a generous gift from Dr. Carlos F. Ibanez (Karolinska Institutet, Stockholm, Sweden).^53^ Transfection of cultured hippocampal neurons with the hRet51-GFP construct was performed by the calcium phosphate coprecipitation method. In brief, 2 *μ*g (for morphological studies) or 7.5 *μ*g (for biochemical analysis) of plasmid DNA were diluted in Tris-EDTA (TE) pH 7.3 and mixed with 2.5 M CaCl_2_. This DNA/TE/calcium mix was added to 10 mM HEPES-buffered saline solution (270 mM NaCl, 10 mM KCl, 1.4 mM Na_2_HPO_4_, 11 mM dextrose and 42 mM HEPES, pH 7.2). The precipitates were allowed to form for 30 min at room temperature (RT), protected from light, with vortex-mixing every 5 min, to ensure that the precipitates had similar small sizes. Meanwhile, cultured hippocampal neurons were incubated in culture-conditioned medium with 2 mM kynurenic acid. The precipitates were added dropwise to each well and incubated for 90–120 min at 37 °C in an incubator with 95% air/5% CO_2_. The cells were then washed with acidic culture medium containing 2 mM kynurenic acid and returned to the 95% air/5% CO_2_ incubator for 20 min at 37 °C. Finally, the medium was replaced with the initial culture-conditioned medium, and the cells were further incubated in a 95% air/5% CO_2_ incubator for 48 h at 37 °C to allow protein expression. Cell cultures were then subjected to OGD for 90 min, and 12 h after the insult the cells were fixed to proceed with the cell death assay. In the case of calpain-induced cleavage of Ret51, hippocampal neurons were subjected to excitotoxic stimulation, in presence or in the absence of the calpain inhibitor MDL28170 (50 *μ*M) or the proteasome inhibitor *β*-lactone (1*μ*M), and further incubated in culture-conditioned medium for the indicated periods of time.

### Cell death assay

Hippocampal neurons were cultured on poly-d-lysine-coated glass coverslips and cell death was evaluated based on the analysis of nuclear morphology. Neurons were fixed in 4% sucrose/paraformaldehyde for 15 min at RT before incubation with the fluorescent dye Hoechst 33342 (1 *μ*g/ml) for 10 min. The coverslips were then mounted with a fluorescent mounting medium (Dako, Glostrup, Denmark), and imaging was performed on a Zeiss Axiovert 200 fluorescence microscope (Jena, Germany), under a × 40 objective. For each experimental condition, three coverslips were used (at least 200 cells per coverslip were counted), and at least three independent experiments were analyzed.

When hippocampal neurons were transfected with hRet51-GFP, following OGD they were fixed in 4% sucrose/paraformaldehyde (in phosphate-buffered saline (PBS)) and permeabilized with 0.3% Triton X-100 in PBS. The neurons were then incubated with 10% BSA in PBS for 30 min at 37 °C, and incubated with the rabbit anti-GFP antibody diluted in 3% BSA in PBS, overnight at 4 °C. The cells were washed with PBS and incubated with the secondary antibody (anti-rabbit IgG) conjugated with Alexa Fluor 488, for 1 h at RT. The coverslips were mounted in a fluorescence mounting medium and imaging was performed on a Zeiss Axiovert 200 fluorescence microscope, under a × 40 objective. The cells to count were chosen by the GFP (green) channel to check for the presence of transfected neurons. Measurements were performed in triplicate (⩾80 cells were counted per coverslip), and in 5–6 independent experiments with distinct preparations.

### Western blot

Hippocampal neurons (7 or 15 DIV) were washed twice with ice-cold PBS and then lysed in RIPA buffer (150 mM NaCl, 50 mM Tris-HCl, pH 7.4, 5 mM EGTA, 1% Triton, 0.5% DOC and 0.1% SDS at a final pH 7.5) supplemented with a mixture of protease inhibitors (0.1 mM PMSF and CLAP (1 *μ*g/ml chymostatin, 1 *μ*g/ml leupeptin, 1 *μ*g/ml antipain and 1 *μ*g/ml pepstatin)) and phosphatase inhibitors (1.5 mM Na_3_VO_4_ and 50 mM NaF). After centrifugation at 16 100 × *g* for 10 min, protein levels present in the supernatants were quantified using the BCA method (Thermo Fisher Scientific). Before separation by SDS-PAGE, in 7.5% or 10% polyacrylamide gels, the samples were denatured with 2x concentrated denaturing buffer (125 mM Tris, pH 6.8, 100 mM glycine, 4% SDS, 200 mM DTT, 40% glycerol, 3 mM sodium orthovanadate and 0.01% bromophenol blue) at 95 °C for 5 min. Following electrophoresis, proteins were transferred to PVDF membranes (Millipore) and were immunoblotted. Membranes were incubated with primary antibodies (overnight at 4 °C or 1 h at RT), washed and exposed to alkaline phosphatase-conjugated secondary antibodies (1 : 20 000 dilution; 1 h at RT). Alkaline phosphatase activity was visualized by enhanced chemifluorescence on the Storm 860 gel and blot imaging system and was quantified using the ImageQuant program (GE Healthcare). Anti-synaptophysin and anti-*β*-tubulin antibodies were used as loading controls.

### Immunoprecipitation

Hippocampal neuronal cultures (15 DIV) were washed twice with ice-cold PBS and then lysed in RIPA buffer supplemented with a mixture of protease and phosphatase inhibitors. After centrifugation at 16 100 × *g* for 10 min, protein levels in the supernatants were quantified using the BCA method. Protein G sepharose beads (50 *μ*l) were added to lysis buffer (1 ml) containing 2 *μ*g of the anti-Ret51 antibody and incubated overnight on a head-over-head shaker at 4 °C. Antibody excess was removed by two rinses with lysis buffer. Lysed samples (700/800 *μ*g) were added to the complexes beads–antibody and incubated for 3 h on a head-over-head shaker at 4 °C. Beads were centrifuged at 800 × *g* and the supernatant was collected, and the complexes beads–antibody–samples were then washed three times with lysis buffer. The residual lysis buffer was removed and then 50 *μ*l of 2 × loading buffer was added. Samples were heated at 95 °C for 5 min and beads were centrifuged at 800 × *g*. The bead supernatants were used for western blot analysis.

### Middle cerebral artery occlusion

Experiments were conducted in accordance with protocols approved by the Malmö/Lund Ethical Committee for Animal Research (M332-09, M243-07). C57BL/6J male mice (11–34 weeks old; weight: 23.0–35 g; Lund University breeding facility) were housed under diurnal conditions with *ad libitum* access to water and food before and after surgery.

Focal cerebral ischemia was induced by transient occlusion of the right MCA as described previously.^54^ In brief, mice were anesthetized by inhalation of 2.5% isoflurane (IsobaVet, Schering-Plough Animal Health) in O_2_:N_2_O (30 : 70). Anesthesia was subsequently reduced to 1.5–1.8% isoflurane and sustained throughout the occlusion period. Body temperature was kept at ~37 °C throughout the surgery period. To monitor regional cerebral blood flow (rCBF), an optical fiber probe (Probe 318-I, Perimed, Järfälla, Sweden) was fixed to the skull at 2 mm posterior and 4 mm lateral to bregma, and connected to a laser Doppler flow meter (Periflux System 5000, Perimed). A filament composed of 6-0 polydioxanone suture (PSD II, Ethicon, Norderstedt, Germany) with a silicone tip (diameter of 225–275 *μ*m) was inserted into the external carotid artery and advanced into the common carotid artery. The filament was retracted, moved into the internal carotid artery and advanced until the origin of the MCA, given by the sudden drop in rCBF (~70% of baseline). After 45 min, the filament was withdrawn and reperfusion was observed. The animals were placed in a heating box at 37 °C for the first 2 h after surgery and thereafter transferred into a heating box at 35 °C to avoid postsurgical hypothermia. Thirty minutes and 24 h after the onset of reperfusion, 0.5 ml of 5% glucose was administered subcutaneously. Temperature and sensorimotor deficits were assessed at 1, 2 and 24 h after the surgery. Body weight was controlled daily. In sham surgeries, the filament was advanced up to the internal carotid artery and was withdrawn before reaching the MCA. After (permanent) ligation of the external carotid artery, 0.150 ml of 0.05% bupivacaine (Marcain, AstraZeneca, Södertälje, Sweden) were injected around the wound to reduce pain. Mice that did not verify an immediate reduction in rCBF upon MCAO or reperfusion were immediately killed and excluded from the study; intracerebral hemorrhage was confirmed a *posteriori* in all cases.

Mice were anesthetized 48 h after MCAO or sham surgery, by inhalation of 2.5% isoflurane, and were then perfused transcardially with 0.9% NaCl for 2 min before decapitation. Upon removal of meninges, brains were rapidly isolated and frozen by immersion in isopentane at −40 °C, further cooled down to −70 °C and stored at −80 °C. The infarct core and remaining ipsilateral tissue (designated as penumbra for simplification) were dissected, as well as the contralateral cortex, from coronal brain sections covering the majority of damage. More specifically, consecutive 2-, 1- and 2-mm-thick brain sections were made, starting at 2 mm from the olfactory bulb. Dissections were performed at −15 °C, a temperature that allows an easy detachment of the infarct core and penumbra. The cortical–striatal infarcts obtained were as previously illustrated.^38^ Equivalent brain regions were dissected from sham-operated mice, which were also designated as infarct core and penumbra, and contralateral cortex. For each animal, corresponding regions from each of three consecutive brain sections were pulled together. Samples were then homogenized and processed for western blotting as previously described.^38^ Cellular protein extraction was performed by mechanical homogenization of the tissue and incubation in lysis buffer: 20 mM Tris (pH 7.5), 150 mM NaCl, 1 mM EDTA, 1 mM EGTA, 1% Triton X-100, 2.5 mM sodium pyrophosphate, 1 mM *β*-glycerolphosphate, 1 mM orthovanadate and 1 mM PMSF, supplemented with a protease inhibitor cocktail. Following 30min incubation at 4 °C, samples were centrifuged at 18 000 × *g* for 15 min. Total protein concentration in lysates was determined by the Bradford assay.

### 2,3,5-Triphenyltetrazolium chloride staining

After 24 h of recovery, animals were sacrificed in order to evaluate the infarct volume. The brain was removed and the forebrain was sliced into 1 mm thick sections using a mouse brain slicer, on ice. The sections were rinsed once in ice-cold 0.9% sodium chloride (NaCl) for 10 min and subsequently immersed in 50 ml of 0.01% 2,3,5-triphenyltetrazolium chloride in 0.9 % NaCl at 37 °C for 15 min. Slices were fixed in 4% formalin and images were acquired with a MicroPublisher 3.3 RTV CCD camera (QImaging, Surrey, BC, Canada) using standard conditions.

### Statistical analysis

Statistical analysis was performed using one-way ANOVA analysis of variance, followed by Dunnett's or Bonferroni's *post hoc* test, or using Student's *t*-test, as indicated in the figure captions.

## Abbreviations

4-AP: 4-aminopyridine
*β*-lactone: *clasto*-lactacystin *β*-lactone
ALLN: N-acetyl-Leu-Leu-Norleu-al
APV: DL-2-amino-5-phosphonovaleric acid
CNQX: 6-cyano-7-nitroquinoxaline-2,3-dione
DIV: days in vitro
GDNF: glial cell line-derived neurotrophic factor
GFP: green fluorescent protein
GFR*α*1: GDNF family receptor alpha-1
GPS-CCD: group-based prediction system-calpain cleavage detector
MAPK: mitogen-activated protein kinase
MCAO: middle cerebral artery occlusion
MDL28170 (MDL): carbobenzoxy-valinyl-phenylalaninal
MK-801: (5*R*,10*S*)-(+)-5-Methyl-10,11-dihydro-5*H*-dibenzo[*a*,*d*]cylcohepten-5,10-imine maleate
NMDA: N-methyl-d-aspartate
NMDAR: N-methyl-d-aspartate receptor
OGD: oxygen and glucose deprivation
PLC: phospholipase C
PMSF: phenylmethylsulfonyl fluoride
SBDP: spectrin breakdown product
