# Supplementary material for: Brain ischemia downregulates the neuroprotective GDNF-Ret signaling by a calpain-dependent mechanism in cultured hippocampal neurons
Source: Cell Death Dis. 2015 Feb 12;6(2):e1645–. doi: 10.1038/cddis.2014.578 (PMC4669807; doi:10.1038/cddis.2014.578)
Supplement: Supplementary Information [file cddis2014578x1.doc]

**Supplementary information**

**Supplementary methods**

**Preparation of extracts for proteasome activity**

Hippocampal neurons were washed twice with ice-cold phosphate buffered saline buffer (PBS). The cells were then lysed in 1 mM EDTA, 10 mM Tris–HCl pH 7.5, 20% glycerol, 4 mM dithyothreitol (DTT) and 2 mM ATP (100 μl/well). After sonication and centrifugation at 16 100 x *g* for 10 min at 4 °C, total protein content in the supernatant was quantified using the Bio-Rad Protein assay, and the concentration of the samples were equalized with lysis buffer.

**Proteasomal peptidase activity assays**

Peptidase activities of the proteasome were assayed by monitoring the production of 7-amino-4-methylcoumarin (AMC) from the fluorogenic peptide Suc-LLVY-AMC. Samples (20 μg) were incubated with the fluorogenic substrate, 25 μM Suc-LLVY-AMC, in 25 mM Tris–HCl (pH 8.0) and 0.5 mM EDTA buffer, in a final volume of 100 μl. The release of fluorescent AMC was measured at 37 °C using a microplate reader SPECTRAmax Gemini EM at an excitation wavelength of 360 nm and an emission wavelength of 460 nm, for 60 min, at 5 min intervals. All the experiments were performed in the presence of 2 mM ATP. Specific activity was determined by subtracting the activity measured in the presence of 10 μM MG132, a proteasome inhibitor.

**Supplementary figure legend**

**Figure S1. Effects of proteasome inhibition by β-lactone in hippocampal neurons.** (**a**-**c**) Cultured hippocampal neurons (7 DIV) were incubated with 0.05, 0.1 or 1 μM β-lactone during 6 h. (**a**) The chymotrypsin-like activity was measured with the fluorogenic substrate 25 μM Suc-LLVY-AMC. The results are the average ± S.E.M. of 3 different experiments performed in independent preparations. Statistical analysis was performed by one-way ANOVA, followed by Dunnett's test (****P*<0.001, ***P*<0.01). (**b**) The accumulation of ubiquitinated proteins was determined by western blotting, using an antibody anti-ubiquitin (**b**) and the quantification of the results obtained for the 75 kDa-250 kDa proteins is shown in (**c**). The control condition, corresponding to neurons not exposed to the proteasome inhibitor, was set to 100%.

**Figure S2. GDNF signaling machinery in 15 DIV cultured hippocampal neurons.** (**a**) Hippocampal neurons at 7 and 15 DIV were stimulated or not with 10 ng/ml GDNF (GDNF) and cell extracts were analyzed by western blotting with a phospho-specific Ret antibody (pRet, Y1062). The ratio between pRet (Y1062) protein levels and the loading control (tubulin) was calculated and pRet expression in control unstimulated 15 DIV neurons was set to 100%. (**b**) Hippocampal neurons (15 DIV) were stimulated with 10 ng/ml GDNF (GDNF) under control condition and after excitotoxic stimulation with glutamate (Glu; 50 μM, 20 min). Under the latter experimental conditions the cells were incubated in culture-conditioned medium for 6 h after the toxic insult before stimulation with GDNF. When the effect of the calpain inhibitor MDL28170 (MDL; 50 μM) was tested, the cells were pre-incubated with the inhibitor for 2 h before glutamate stimulation, and the inhibitor was also present during all additional experimental manipulations. Cell extracts were analyzed by western blotting with anti-Ret51 (intracellular epitope) antibody. The ratio between Ret51 protein levels and the loading control (tubulin) was calculated and the control Ret51 protein level was set to 100%. (**c**, **d**) Cultured hippocampal neurons (15 DIV) were subjected to excitotoxic stimulation with glutamate (50 µM glutamate, 20 min) and further incubated in culture-conditioned medium for 2-8 h. Cell extracts were analyzed by western blotting using anti-Ret51 (intracellular epitope) (**c**) or anti-Ret9 (intracellular epitope) (**d**) antibodies at the indicated time points after excitotoxic stimulation. The ratio between Ret51 (**c**) or Ret9 (**d**) and the loading control (tubulin) was calculated and the results obtained under resting conditions were set to 100%. (**a**-**d**) The results are the average ± S.E.M. of 4-6 different experiments performed in independent preparations. Statistical analysis was performed using one-way ANOVA followed by (**a**, **b**) Bonferroni’s multiple comparison test (NS, not significant; ****P*<0.001, ***P*<0.01 as compared with the control or for the indicated comparisons; ^###^*P*<0.001, as compared with GDNF stimulated cells under control conditions) or by (**c**, **d**) Dunnett’s comparison test performed for each condition as compared with the control, not exposed to excitotoxic conditions (NS, not significant; ****P*<0.001; Ret9 not significantly different when compared with the control protein levels).
