# Supplementary figures and images for: Brain ischemia downregulates the neuroprotective GDNF-Ret signaling by a calpain-dependent mechanism in cultured hippocampal neurons
Source: Cell Death Dis. 2015 Feb 12;6(2):e1645–. doi: 10.1038/cddis.2014.578 (PMC4669807; doi:10.1038/cddis.2014.578)

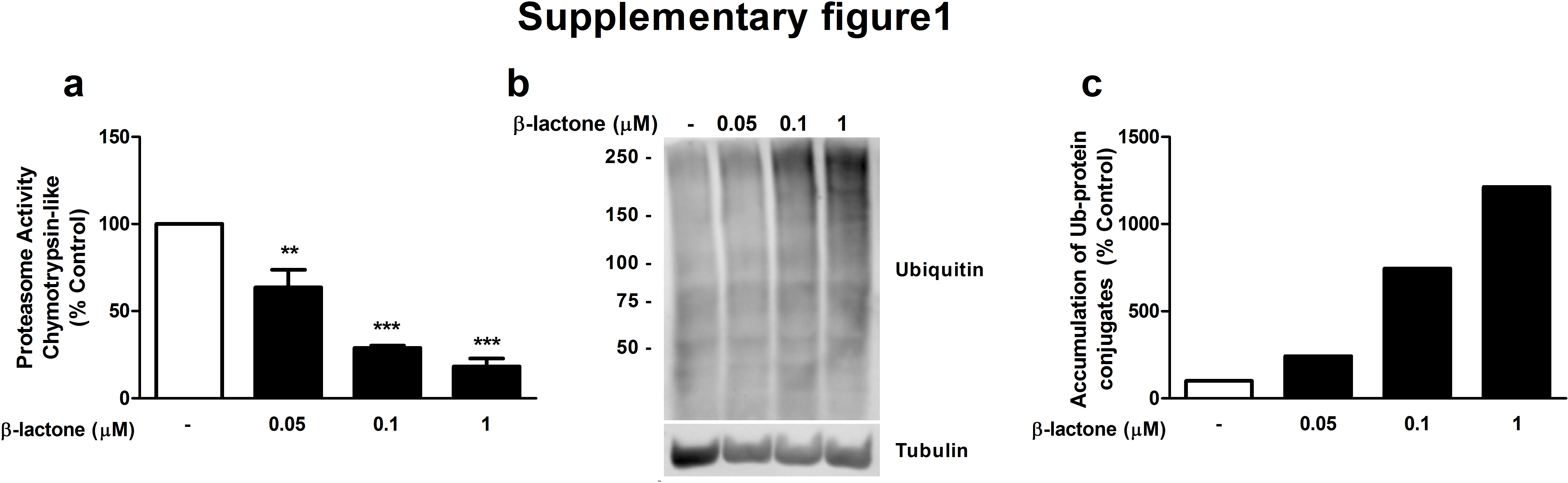

Supplement: Supplementary Figure 1 [file cddis2014578x2.tif]

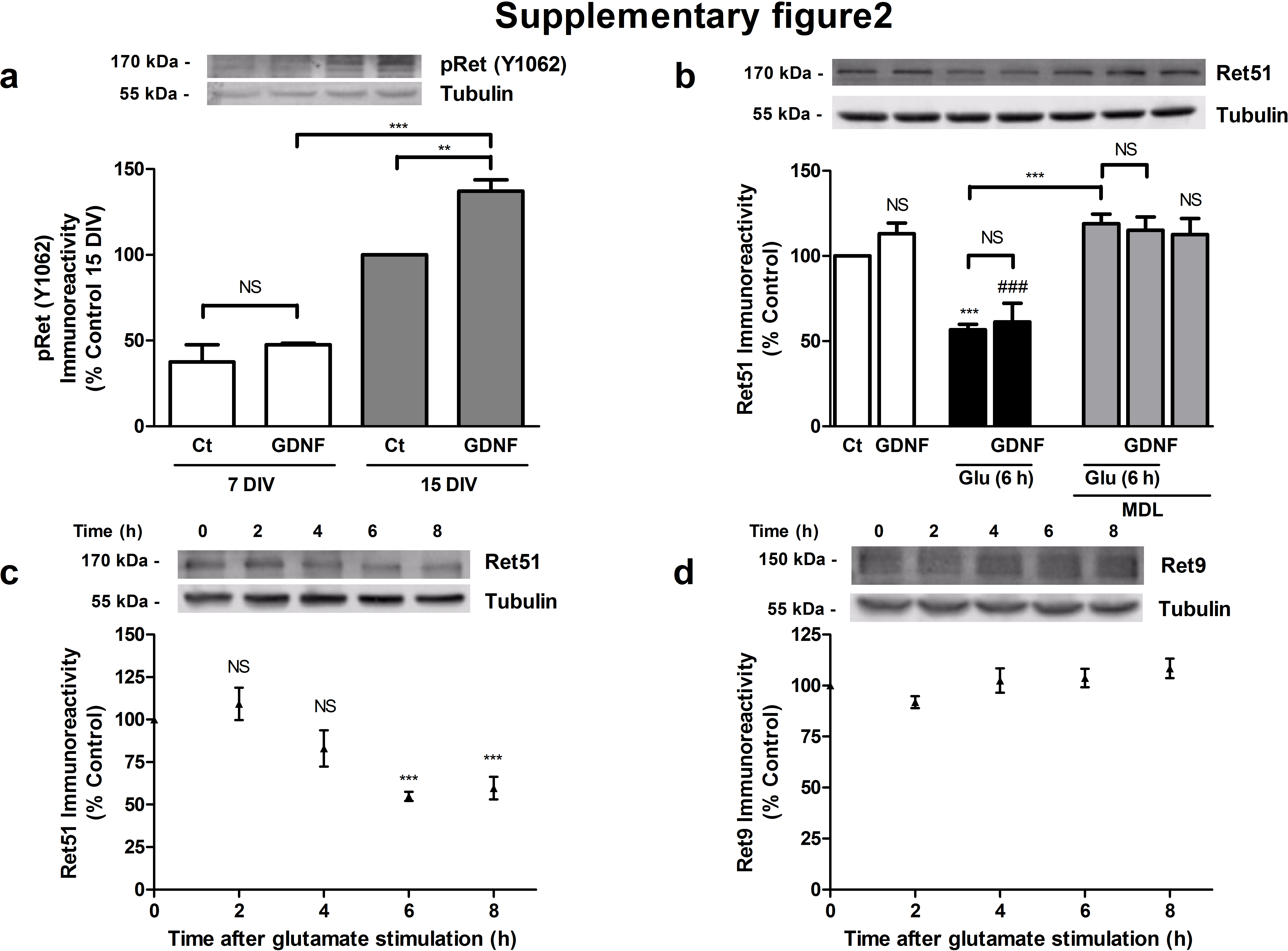

Supplement: Supplementary Figure 2 [file cddis2014578x3.tif]
